# Reconfiguring Nature’s Cholesterol Accepting Lipoproteins as Nanoparticle Platforms for Transport and Delivery of Therapeutic and Imaging Agents

**DOI:** 10.3390/nano10050906

**Published:** 2020-05-08

**Authors:** Skylar T. Chuang, Siobanth Cruz, Vasanthy Narayanaswami

**Affiliations:** Department of Chemistry and Biochemistry, California State University, Long Beach, 1250 Bellflower Blvd, Long Beach, CA 90840, USA; stc89@scarletmail.rutgers.edu (S.T.C.); scruz188@gmail.com (S.C.)

**Keywords:** apolipoprotein AI, apolipoprotein E, nanodiscs, reconstituted HDL, lipoproteins, bioflavonoids, gold nanoparticles, drug delivery, cancer therapy, diagnostics

## Abstract

Apolipoproteins are critical structural and functional components of lipoproteins, which are large supramolecular assemblies composed predominantly of lipids and proteins, and other biomolecules such as nucleic acids. A signature feature of apolipoproteins is the preponderance of amphipathic α-helical motifs that dictate their ability to make extensive non-covalent inter- or intra-molecular helix–helix interactions in lipid-free states or helix–lipid interactions with hydrophobic biomolecules in lipid-associated states. This review focuses on the latter ability of apolipoproteins, which has been capitalized on to reconstitute synthetic nanoscale binary/ternary lipoprotein complexes composed of apolipoproteins/peptides and lipids that mimic native high-density lipoproteins (HDLs) with the goal to transport drugs. It traces the historical development of our understanding of these nanostructures and how the cholesterol accepting property of HDL has been reconfigured to develop them as drug-loading platforms. The review provides the structural perspective of these platforms with different types of apolipoproteins and an overview of their synthesis. It also examines the cargo that have been loaded into the core for therapeutic and imaging purposes. Finally, it lays out the merits and challenges associated with apolipoprotein-based nanostructures with a future perspective calling for a need to develop “zip-code”-based delivery for therapeutic and diagnostic applications.

## 1. Introduction and Historical Perspective

Lipoproteins are large lipid/protein complexes that have been nature’s simple and elegant solution to the problem of transporting lipophilic biomolecules in the aqueous environment of biological systems. The notable unifying feature of the cargoes is their nonpolar or amphipathic nature that includes a broad range of endogenously occurring molecules such as cholesterol, cholesterylester (CE), triglycerides, fatty acids, vitamins, hormones, and metabolites. Broadly, from a structural perspective, lipoproteins are spherical complexes composed of a monolayer of amphipathic lipids and apolipoproteins (apo) that encapsulate a core of neutral or nonpolar lipids. In humans, there are several types of lipoproteins that may be classified based on their electrophoretic mobility or density: chylomicron, very low-density lipoprotein (VLDL), intermediate density lipoprotein (IDL), low-density lipoprotein (LDL), and high-density lipoprotein (HDL). They differ in their origin or site of synthesis, composition, and cargo, with chylomicrons originating in the intestines transporting dietary lipids, VLDL synthesized by the liver, IDL and LDL derived from VLDL, and HDL synthesized by macrophages, small intestines, and astrocytes. Whereas all lipoproteins in general have provided guiding principles, HDL in particular, especially the nascent particles that are generated during the biogenesis of HDL (also referred to as pre-β HDL) have served as model systems for the development of apo-based nanoparticles as platforms for transport and delivery exogenous nonpolar/amphipathic molecules such as drugs, therapeutic agents, flavonoids, imaging, and diagnostic molecules.

**Figure 1:**
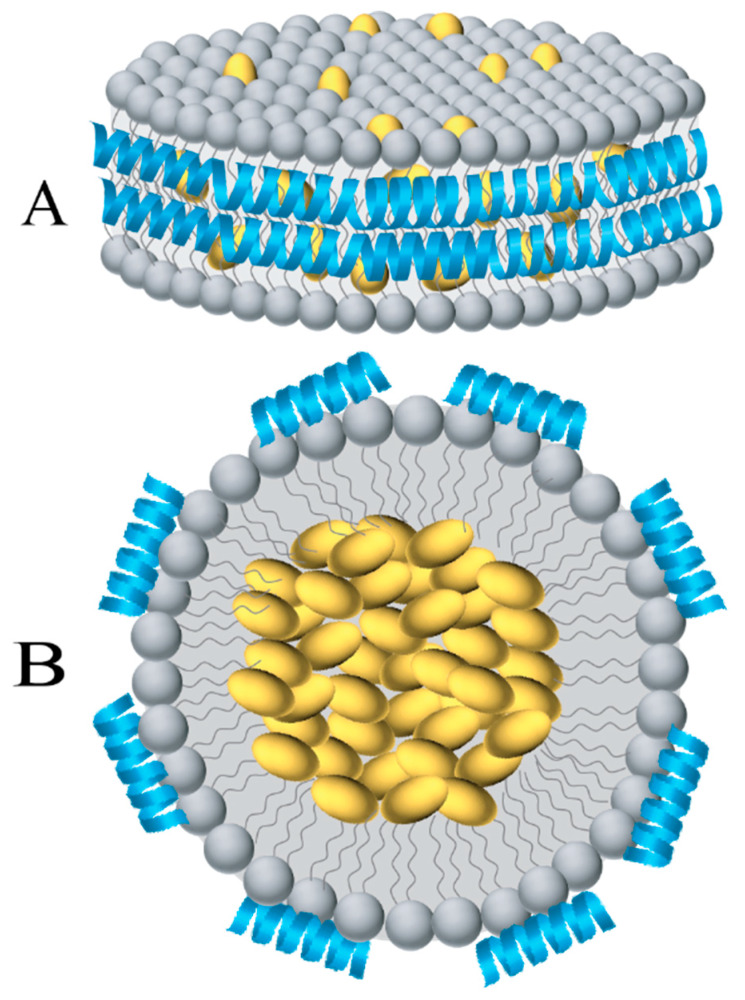
Schematic depiction of discoidal and spherical nanolipoproteins with embedded cargo. (**A**) Discoidal apolipoprotein-based nanostructures (nanodiscs) are composed of a bilayer of phospholipids (grey) with amphipathic α-helices of apolipoproteins (blue) circumscribing the bilayer. (**B**) Cross-section of a spherical lipoprotein composed of a monolayer of phospholipids and small apo-based helical peptides. The phospholipid interior offers an ideal environment to embed hydrophobic and/or amphipathic biomolecules (yellow).

The concept of employing lipoprotein particles for drug encapsulation emerged more than 3 decades ago, when naturally occurring lipoproteins were initially employed to transport therapeutic and diagnostic agents [1]. In early studies, LDL isolated from human plasma was de-cored and substituted with biomolecules (called LDL anchors) with comparable polarity as CE (for example retinyl or oleoyl groups) to obtain reconstituted LDL [2]. Some incorporated lysomotropic agents bearing long-chain alkyl amines that tend to be protonated in an acidic environment such as that found in lysosomes. Subsequently, synthetic phospholipids with or without other lipids were used to generate reconstituted HDL (rHDL) which resemble native HDL (Figure 1). Throughout this review, the term rHDL will be used interchangeably with synthetic HDL, nanolipoproteins- or apolipoprotein-based nanostructures.

A fundamental property of apolipoproteins is their ability to bind and convert vesicular or micellar phospholipid structures to form small discoidal bilayer structures that are soluble and stable. Termed initially as roulettes, bicelles, and, subsequently, as nanodiscs, these discoidal structures (10–20 nm in diameter) were found to be composed of a bilayer of phospholipids (4–5 nm wide) that are surrounded by apolipoproteins in the periphery.

## 2. Structural Organization of the Nanodisc Platform 

Biophysical studies of the discoidal particles carried out in the 1990s laid the groundwork for our current understanding of the structural organization of nanodiscs. Structure–function relationships were carried out with lipid-associated intact, truncated, and fragmented variants and peptide segments of apolipoprotein AI (apoAI), apolipophorin III (apoLp-III), an insect apolipoprotein, and apolipoprotein E3 (apoE3). The predominant secondary structural feature of these proteins in their lipid-free state is the amphipathic α-helix, characterized broadly by arrangement of residues with hydrophobic side chains on one face of the helix and those with polar side chains on the other. Many of these apolipoproteins are further characterized by a specific type of amphipathic helix called class A helix, which bears negatively charged residues in the center of polar face and positively charged residues at the polar/nonpolar interface [3]. This arrangement aids in high efficiency lipid binding of the exchangeable apolipoproteins, with a remarkable ability to dissociate from lipids and enable particle re-modulation in response to changes in lipid composition. The unique structural flexibility has significant physiological relevance when using nanodiscs for various therapeutic purposes since it implies that there may be enable particle remodulation and mixing between lipoprotein components of the plasma if administered intravenously. 

One of the cornerstone studies that enabled our understanding of nanodiscs is the report on the unique spatial orientation of the α-helices of apoLp-III in binary complex with 1,2-dimyristoyl-*sn*-glycero-3-phosphocholine (DMPC) [4]. Native apoLp-III was isolated from the hemolymph of the sphinx moth *Manduca sexta*, where it is found in abundance in a 5-helix bundle lipid-free state, and reconstituted with DMPC, which yielded uniform discoidal particles of ~20 nm diameter and 5 nm width. Based on particle compositional analysis, non-denaturing polyacrylamide gel electrophoresis and electron microscopy data, a model of DMPC/apoLp-III discoidal complexes was presented wherein 4–6 apoLp-III molecules circumscribe a bilayer of phospholipids (later termed the “belt” model) with the α-helices oriented normal with respect to the fatty acyl chains [5]. Further experimental evidence for the perpendicular orientation model was obtained from attenuated total reflectance-Fourier transform infrared (ATR-FTIR) spectroscopic analysis [4]. Although the experiment was carried out using hydrated thin films of DMPC/apoLp-III on a germanium plate (i.e., not in solution), the ATR-FTIR data of the amide I region of apoLp-III (which reflects the secondary structure of a protein) indicated that the axes of the α-helices were parallel to germanium plate and to the plane of the lipid bilayer, i.e., perpendicular to the axis of the fatty acyl chains. Upon independently assessing the orientation of phospholipid acyl chains with respect to the germanium surface, it was inferred that the lipid acyl chains were oriented perpendicular to the helical axes of apoLp-III. Further, using metrics of a typical α-helix, it was calculated that two helices would be required to prevent exposure of the fatty acyl chains to the aqueous medium. The conformational change in apoLp-III from a lipid-free helix bundle to lipid associated extended helix configuration is analogous to that of foldable tent poles wherein a compact bundle can be converted to a long, bendable, curved, extended pole. The apolipoprotein component forms the framework and scaffold that maintains the structural integrity of the nanoparticles, similar to that noted in naturally occurring lipoproteins.

This report challenged other studies with DMPC/apoAI [6] and 1,2-palmitoyl-*sn*-glycero-3-phosphocholine (DPPC)/apoAI [7] (wherein apoAI isolated from human plasma was used [8]) and DMPC/apoE3 or its isolated thrombolytic fragments, DPPC/apoE3 or its isolated thrombolytic fragments [9]. These studies had also employed ATR-FTIR and fluorescence spectroscopic analyses and had presented a model wherein the amphipathic α-helices of the lipid-associated apolipoproteins were oriented parallel to the phospholipid fatty acyl chains, resembling a “picket fence”. Although attractive, in light of the sequence-based algorithms that predict Pro punctuations among 22 residue helical segments (that could span a phospholipid bilayer) [3] and since the proposed topology was in agreement with theoretical modeling of apoAI at lipid/water interface [6], this model did not receive further consideration with mounting evidence in support of the “belt” model. Nevertheless, interest in the picket fence model appears to be resurfacing with peptide mimetics appearing to adopt a parallel orientation with respect to the fatty acyl chains [10]. The “belt” model received validation from other studies conducted with native and truncated apoAI, wild-type, and isolated N- and C-terminal domains of apoE3 that approached the issue from a variety of angles such as ATR-FTIR spectroscopy, fluorescence resonance energy transfer (FRET) and depth quenching analysis, X-ray crystallography, and cross-linking analysis [11,12,13,14]. Interestingly, X-ray crystal analysis of an N-terminal truncated form of apoAI (residues 44–243) in lipid-free state showed that the protein adopted a continuous amphipathic helical configuration resembling a “horseshoe” demarcated by kinks at the Pro residues [15]. In addition to providing structural rationale for a belt configuration, this study suggested a predisposition for selected segments of apoAI (and perhaps other apolipoproteins bearing similar amphipathic helical segments) to adopt an extended and curved helical structure when presented with an appropriate environment.

The implication for the belt-like organization and flexibility of apolipoprotein structure in nanodiscs is that it would it allow loading of or substitution of existing phospholipids by hydrophobic molecules, Figure 1. The former is envisaged as small HDL (~10 nm diameter) in which apoAI bears “looped out” flexible segments in lipid associated state as noted in hydrogen deuterium exchange coupled to mass spectrometry studies [16]. By increasing the lipid: protein ratio during reconstitution, the particle diameter can be manipulated [17]. While reconstituting nanodiscs with nonpolar molecules that do not resemble phospholipid structures is possible, one can predict packing defects that result in nanodiscs that are less discrete, as noted in loading with polyphenols [18] (described below). Such nanodiscs may be more prone to rapid exchange of components and interconversion with endogenous lipoproteins compared to compact empty nanodiscs. In early studies, the nanodiscs were adapted for two major purposes:

### 2.1. Structural Analysis of Membrane Proteins

One of the earliest adaptations of lipoproteins is the employment of the soluble nanodiscs encircled by a belt of membrane scaffold protein (MSP) as platforms to study integral membrane proteins (the reader is referred to several excellent reviews in References [19,20,21,22,23,24,25,26]). The MSP is an engineered variant of apoAI that serves as a scaffold and is composed of 22-mer Pro and Gly-punctuated amphipathic α-helical repeats with the helix designations based on the assignment described by Brouillete et al. [27]. The nanodiscs lend themselves well to study membrane proteins in their native environment with access from both sides of the bilayer, unlike a liposome or detergent micelle. The nanodisc technology takes advantage of the ability of MSP and phospholipids to self-assemble in the presence or absence of detergents, during which process membrane proteins could be incorporated. By varying the length of the MSP by truncation and extension, and the nature and content of phospholipids (DMPC, DPPC, 1-palmitoyl-2-oleoyl-glycero-3-phosphocholine (POPC)), the diameter of the nanodisc can be engineered to a desired length [24]. The nanodisc platform offers a large bilayer area (4000–9000 Å^2^) wherein the target membrane protein may be embedded, with displacement of some phospholipids a likely possibility.

### 2.2. Acceptors of Cholesterol Efflux for Improved Cardiovascular Outcomes

The development of empty nanodiscs for use as platforms to embed various types of lipophilic cargo derived inspiration from physiologically occurring events involving HDL. HDL is considered a critical antiatherogenic factor due to the fact of its involvement in the process called reverse cholesterol transport, where it serves as an acceptor and transporter of cholesterol effluxed from peripheral tissues including macrophages to the liver for eventual disposal as bile [28]. This process involves the initial formation of nascent discoidal HDL as a result of ATP-binding cassette A1 (ABCA1) mediating cholesterol efflux [28]. In turn, the nascent HDL thus generated is an efficient acceptor of cholesterol via ABCG1 and is subsequently converted enzymatically to spherical HDL with a core-bearing lipids such as CE. They are eventually responsible to transport cholesterol back to the liver for selective uptake of CE via the cell surface localized scavenger receptor class B type 1 (SR-B1) also referred to as the “HDL receptor” (discussed below). The apoAI on HDL serves as a ligand for the SR-B1. While there were intense efforts to increase HDL levels initially as per the “HDL hypothesis” (raising plasma HDL levels may reduce risk of cardiovascular disease), the turn of the millennium witnessed a trend to increase the functionality and quality of HDL rather than merely raising HDL levels. The reconfigured form of nascent HDL was assembled from various types of phospholipids, some with sphingomyelin, and with apolipoproteins to form rHDL, for developing their use in treatment of cardiovascular disease. Specifically, apoAI and derivatives of apoAI (including the mutant form apoAI_Milano_ bearing R173C, which was inspired by the naturally occurring genetic variant that afforded protection against cardiovascular disease) have received intense scrutiny over the last two decades in efforts to improve cardiovascular outcomes in human trials [28,29,30,31,32,33,34,35,36]. Other mutant apoAI, such as V156K, also show potential as anti-inflammatory and anti-atherosclerotic agents in vitro and in vivo [37]. It was recognized that although the synthetic nanodiscs mimic nascent HDL in morphology and cholesterol efflux ability and bear potential for drug delivery, they may not recapitulate the full pleiotropic effects associated with mature HDL, including anti-oxidative, antithrombotic, and anti-inflammatory activities [38]. 

## 3. The Protein Scaffold of Apolipoprotein-Based Nanostructures

Many different apolipoproteins and lipoproteins have been employed to develop multicomponent synthetic discoidal and spherical lipoproteins (Table 1).

### 3.1. Repurposed LDL and its Derivatives

**Figure 2:**
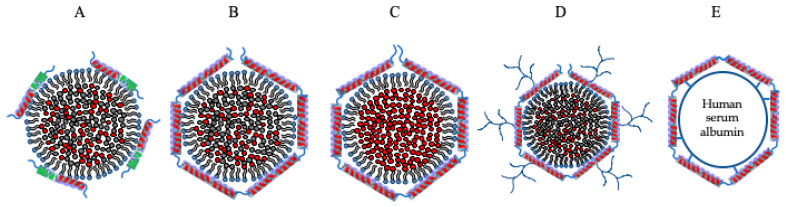
Apolipoprotein-based nanostructures targeting LDLr family of proteins. Nanoparticles bearing ligands, such as apoB-100 or apoE3 or their derivatives, target the LDLr family of proteins. (**A**) ApoB-100-based peptides with a hydrophobic tail to promote lipid binding. (**B**) Plasma-derived LDL bearing apoB-100 on the surface and a core of neutral lipids such as CE or triglycerides with hydrophobic molecules incorporated into the core. (**C**) The core of the LDL particle has been substituted with hydrophobic agents of interest with a surrounding monolayer of amphipathic lipids and apoB-100. (**D**) ApoB-100 on LDL modified with dextran or dendrimers. (**E**) ApoE crosslinked to human serum albumin (HSA) nanoparticles. Particle sizes are not to scale.

Early studies focused on repurposing LDL derived from human plasma or LDL-protein apoB-100 and its derivatives as platforms for incorporating drugs. LDL is a large lipid–protein complex (~25 nm diameter) composed of a core containing neutral lipids, such as CE, and a limited number of triglycerides, surrounded by a monolayer amphipathic lipids, such as phospholipids and cholesterol, and a single, large, non-exchangeable apolipoprotein, apoB-100 (~4500 residues, ~500 kDa). ApoB-100 serves as a scaffold and maintains the integrity of the dynamic LDL, by wrapping around the entire particle. A segment of apoB-100 rich in basic residues towards the C-terminal end (3359-3367) serves as a ligand for direct interaction with the LDL receptor (LDLr), which internalizes the particle by receptor-mediated endocytosis. The internalized particle goes through the endosomal/lysosomal pathway, with the contents eventually released due to the acidic environment lysosomal compartments. Figure 2 shows apolipoproteins used to target the LDLr family of proteins, including apoB-100 and apoE3 (described below). This approach received favorable attention with the early identification that cancer cells overexpress LDLr in order to recruit and capture exogenous sources of cholesterol for their rapid proliferative and metastatic activities (see [50] and references therein). Some examples include:

(a) A minimalist approach wherein an apoB-100-derived peptide bearing the LDLr binding segment [67,68,69,70] was developed to form a synthetic nano-LDL (discussed in Section 3.5).

(b) Use plasma-derived LDL for incorporating drugs as such [50,51,52,53,54,55] or following incorporation of cholesterol–carborane conjugates into LDL for boron neuron capture therapy [71] or derivatized to improve lipophilic nature [72]. Other strategies include use of photosensitizers for controlled release of fluorescent dye for improved cytosolic release by photochemical internalization [55]. In this approach, the dye was intercalated in the phospholipid monolayer of LDL (surface-loaded) or covalently bound to amino acid residues on apoB-100 (protein loading) or incorporated by reconstitution into the nonpolar core of LDL (core-loaded); following cellular internalization, laser irradiation triggered release of the surface-loaded and protein-bound dyes but not core-loaded dyes.

(c) Employment of plasma-derived LDL to de-core existing neutral lipid interior by solvent or detergent extraction or by enzymatic means and replacement with cytotoxic or chemotherapeutic agents upon reconstitution back with apoB-100 [2,50,56,57,58]. A variation of the de-coring approach involves re-assembling the LDL particle using synthetic lipids with incorporated drugs and solubilized apoB-100 [59,60]. Further, LDL has been used for computed tomography (CT) imaging by incorporating gold nanoparticles [61] or by replacing existing lipids with poly-iodinated triglycerides and reconstituting with apoB-100 [62].

(d) Derivatization of plasma-derived LDL by surface coating with polar moieties such as dextran, a polysaccharide used as drug carrier shows promise since it increases the surface area for drug loading without compromising the LDLr binding capability of the lipoprotein [73]. Conjugation of LDL with drug-conjugated poly(propylene imine) dendrimers, bearing multiple generations of branching [74] offers a similar proposition for transporting polar drugs conjugated to the dendrimer in the corona layer or for embedding nonpolar drugs into the particle core, with the dendrimers serving as a protective covering against rapid proteolytic degradation.

### 3.2. ApoAI and ApoE3

Given the long history of apoAI-based nanodiscs with an emphasis on understanding the structure and molecular organization of the protein component, ease of preparation and scalability with native or recombinant protein [75], tolerability in human clinical trials [76], and the optimal size of particles (~10 nm diameter), apoAI (243 residues) has been the apolipoprotein of choice for employment as a drug delivery platform [77,78,79,80,81,82,83]. It is a major exchangeable apolipoprotein component of HDL and is composed of an N-terminal domain organized as a helix bundle and a C-terminal domain that undergoes a concentration-dependent fluctuation between an unstructured and helical state [84]. In its lipid-free (also referred to as lipid-poor) state, apoAI plays a major role in promoting lipid efflux via ABCA1 leading to formation of a discoidal HDL particle composed predominantly of phospholipids [85]. 

The main point of entry for apoAI-based lipoproteins would be SR-B1 which is also known to mediate bidirectional flux of CE [86]. Recent observations that (similar to LDLr) SR-B1 is overexpressed in many cancer cell types [87,88,89], makes it an attractive candidate for drug targeting [80,90]. SR-B1 is a dimeric protein with the monomer composed of two transmembrane and a large extracellular domain with the N- and C-terminal ends facing the interior of the cell. It binds HDL with high affinity via its extracellular domain which is juxtaposed to the cell membrane surface. The amphipathic α-helix in apoAI (or its CNBr cleaved fragments), the N- and C-terminal apoAII, apoE, apoCIII or a model peptide of amphipathic class A helix is the recognition motif for direct protein–protein interaction with and binding to SR-B1, when presented as a nanodisc or as spherical HDL3 or as lipid-free protein [91]. When the ligand has a core as in HDL, docking between the dimeric SR-B1 and HDL is believed to allow diffusion of the core CE into a nonpolar channel in SR-B1 and lateral movement into the membrane bilayer, without uptake of the HDL particle or its remnant or apoAI. Recent studies further define SR-B1-mediated CE extraction as a “nibbling” mechanism wherein a few molecules are extracted from several HDL particles (in contrast to “gobbling” wherein all CE molecules are extracted from a few HDL particles [92]. In the current context of synthetic lipoproteins loaded with therapeutic cargo, either or both mechanisms would be suitable for drug delivery via SR-B1. However, the observation that lipid-free apoAI (but not apoAII) dissociates from the remnant HDL (which is now enriched in apoAII) after the CE core is given up to the cells has broad implications in terms efficiency of core payload uptake. Inclusion of apoAII in nanodisc formulations along with apoAI (or other apolipoproteins) may offer a refined approach considering that apoAII is more hydrophobic than apoAI [93,94,95,96] and would therefore confer more stability to the lipoprotein particle.

Although its history is shorter than that of apoAI-based nanoparticles, apoE3 has also been a favorable apolipoprotein of choice for lipoprotein-mediated drug delivery [18,63,97]. It has the added benefit of ability to serve as a ligand for the LDLr family of proteins [98,99] to gain entry into cells in addition to its ability to bind and interact with SR-B1. ApoE3 is a 299 residues glycoprotein composed of an N-terminal (NT) domain (residues 1–191) and a C-terminal (CT) domain (residues 200–299) that are linked via a protease sensitive loop [100,101]. The predominant features of the NT domain are the presence of four long amphipathic α-helices (H1, H2, H3, and H4) (PDB ID# 1NFN and 2L7B) that are folded into a 4 helix bundle. The residues involved in interaction with LDLr are located on H4 and its vicinity. Helix H4 (residues 131–164: EELRVRLASHLR**K**LR**K**RLLRDADDLQKRLAVYQA) is dominated by basic residues (underlined) with K143 and K146 (bold) together with R172 (which lies outside H4) playing a critical role in interaction with acidic residues in the cysteine-rich ligand binding repeats LA5-LA5 on LDLr [102,103] and in clusters II, III, and IV of LDLr-related protein 1 (LRP1) [99,104,105] in lipid-associated state.

**Figure 3:**
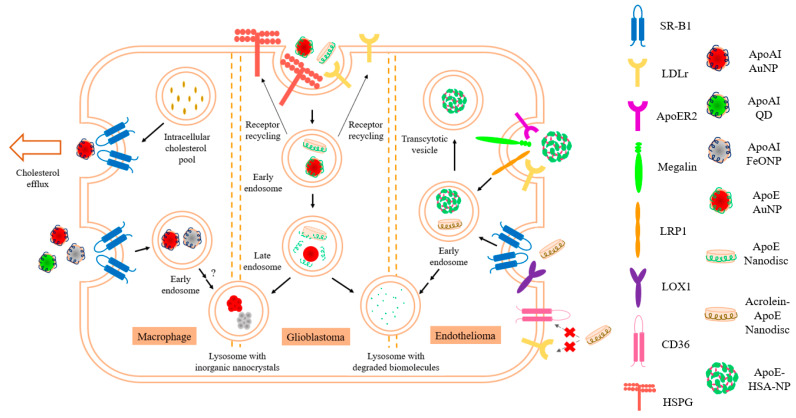
Entry mechanism(s) for various types of nanoparticles (NPs) in cells. HDL reconstituted with apoAI and various types of inorganic nanocrystals are taken up by macrophages, a process that is mediated by SR-B1. HDL-AuNP have been shown to promote cholesterol efflux not only in THP-1 macrophages, but also in other cells that express SR-B1 such as B lymphoma cells and myeloid derived suppressor cells (MDSCs). HDL reconstituted with apoE3, either as discoidal or spherical NPs, could gain entry through the LDLr family of proteins in GBM and possibly also through heparan sulfate proteoglycans (HSPG). In endothelial cells, rHDL reconstituted with acrolein-modified apoE showed entry through both SR-B1 and LOX1, but not through conventional oxidized LDL receptors such as CD36. Interestingly, HSA-crosslinked-apoE3 NP without lipid content were shown to enter brain endothelial cells mainly through LRP-1, although other LDLr family receptors could also contribute to the entry.

The isolated NT domain of apoE3 bearing the LDLr binding sites has the ability to act independent of the CT domain and is sufficient for receptor interaction. Several studies exploited this feature for nanodisc formation [18]; in addition, the isolated domain is more stable, less prone to degradation without the protease-sensitive loop and the CT domain, and can be obtained in substantial yields. Further, the availability of X-ray and NMR structures of apoE3 NT domain (1–191) and (1–183), respectively [106,107], allows a strategic structure-guided approach for developing lipoprotein formulations to target receptors. Its ability to bind SR-B1 [91,108] and/or several core members of the LDLr family of proteins (LDLr, LRP1, LRP1b, LRP2/megalin, VLDL receptor (VLDLr), multiple epidermal growth factor (EGF) repeat containing protein (MEGF7/LRP4), apoE receptor-2 (apoER2)/LRP8)), makes apoE3 an attractive candidate for drug delivery particularly in neurons and cancer cells, which overexpress these receptors [2,81,109,110,111,112,113,114]. This approach was employed to provide proof of concept for cellular uptake and targeting to endosomal/lysosomal sites with flavonoids [63] in glioblastoma multiforme (GBM) cells. The presence of apoE3 on synthetic lipoproteins thus provides a unique ability to target lysosomal storage disorders and others that involve deficiencies in lysosomal enzymes to deliver recombinant enzymes of interest as an embedded enzyme or as a chimera [115]. Figure 3 summarizes various entry mechanisms for apolipoprotein-based nanoparticles into cells.

In addition, apoE3’s role in cell signaling and signal transduction pathways via the LRP family members [116], and its identification as a ligand for the non-LDLr family member, triggering receptor expressed on myeloid cells 2 (TREM2) [117,118,119] broadens the scope of using apoE3 containing lipoproteins for treating neurodegenerative disorders. Additional considerations include the ability of apoE3 to bind and internalize lipoprotein particles following ionic interactions with cell surface localized heparan sulfate proteoglycans [101] (HSPG), a property unique to apoE3, Figure 3. K143 and K146 in the NT domain play a dominant role in heparin binding in the lipid-associated state of apoE3 as nanodiscs; they bear a much lower pK_a_ value compared to other lysines in the NT domain, and therefore confer a high positive electrostatic potential in their immediate vicinity [120] that allows interaction with HSPG. The HSPG-mediated internalization may occur directly or via transfer to LRP [121].

Further, by chemically and/or oxidatively modifying the apolipoprotein or lipid component of lipoproteins, it would be possible to broaden their entry points and/or reroute them to alternative ports of entry into cells. It is well established that oxidized lipoproteins acquire altered ability to interact and bind scavenger receptors in macrophages, a process that plays a critical role as an early event in atherogenesis and plaque formation [122,123]. In the case of LDL, oxidation or acetylation that lead to modification of apoB-100 and/or the phospholipid or cholesterol, disables its ability to undergo receptor mediated endocytosis via interaction with the LDLr but confers an ability to recognize scavenger receptor class A (SR-A) [123] (Figure 3). In other studies, in vitro modification of plasma HDL by oxidative agents, such as acrolein that modified apoAI and apoAII (and likely other protein and lipid components), increased its ability to bind SR-B1 without a parallel increase in uptake of core components [124]. Recently, we demonstrated that, whereas nanodiscs with unmodified apoE3 gain entry into endothelial cells predominantly via LDLr and to some extent via SR-B1, those with acrolein-modified apoE3 [125] showed significantly decreased ability to bind and be internalized via the LDLr. Instead, they are re-routed through SR-B1 to a larger extent and also become eligible to bind lectin-like oxidized LDL receptor 1 (LOX1) (Figure 3). Taken together, chemical modifications to nanodisc formulations, including acetylation, amine-specific cross-linking, PEGylation, and dendrimer formation, must take into consideration that lysine modifications may redirect the uptake mechanism away from the LDLr to the scavenger receptor family including CD36. Overall, the choice of apolipoprotein drives mode of entry into cells: with apoB-100-derived peptide bearing LDLr binding sites targeting the endosomal and lysosomal compartments by receptor mediated endocytosis, apoAI targeting non-endosomal and non-lysosomal sites and apoE3 targeting both [81,126]. 

Some aspects that require careful consideration are that, although LDLr and SR-B1 are overexpressed in tumor cells, they are expressed in other cells as well, which diminishes the targeting ability of apoE3 and apoAI. Further, LDLr and LRP1 are also present as soluble receptors due to the ectodomain shedding [127,128] which may compete with cell surface localized receptors for binding to apolipoprotein-based nanoparticles and thereby reduce drug availability for internalization. This is particularly relevant at the neurovascular junction where the brain endothelial cells lining the blood brain barrier (BBB) express an abundance of LDLr and LRP1, which undergo ectodomain shedding, thereby decreasing the number of functional receptors.

### 3.3. ApoJ 

ApoJ (~80 kDa), also known as clusterin, is another important constituent of HDL in the brain; it is a disulfide bonded heterodimeric protein that is a member of the heat shock protein family with chaperone function. It is considered an anti-amyloidogenic protein that binds to and inhibits aggregation of soluble amyloid beta peptide (Aβ), and aids in its clearance [65,66]. Reconstituted HDL composed of phospholipids, cholesterol, and recombinant apoJ (rHDL-rApoJ) form large nanodiscs 25–50 nm diameter [129]. These nanoparticles showed an ability to decrease Aβ fibril formation and to mediate cholesterol efflux from J774 macrophages. Further, upon i.v. injection in APP23 mice, a well characterized mouse model of Alzheimer’s disease and cerebral amyloidosis that show seven-fold overexpression of human mutant amyloid precursor protein, fluorescent rHDL-rApoJ appeared to accumulate in the cranial region, particularly in aged mice with high amyloid load. The observation that rHDL–rApoJ co-localized with fibrillar Aβ in the cerebral microvasculature but not in the brain parenchyma suggests that it did not cross the BBB and, therefore, has potential to treat cerebral amyloidosis and related pathologies [129].

### 3.4. Human Serum Albumin Coated with ApoAI or ApoE3

In an alternate approach, thiolated apoE3 was coated on human serum albumin (HSA) nanoparticles (NPs) via poly(ethylene glycol) (PEG) [130,131,132] yielding NPs that were ~200 nm in diameter (Figure 2); the PEG was functionalized with N-hydroxysuccinimde groups covalently attached to the HSA-NP core and with maleimide groups linked to the thiolated apoE3 coat which allowed presentation of apoE3 as a ligand for the LDLr and related family members (Figure 3). The cellular uptake of the apoE3-modified HSA-NP in brain microvascular endothelial cells occurred via LRP1. Further, following intravenous injection in mice, apoE3-modified HSA-NP were identified in neurons, suggesting transcytosis across the endothelial cells lining the BBB.

The HSA has also been covalently conjugated to apoAI, apoE3 or apoB-100 to deliver loperamide to the central nervous system (CNS) [133,134]. These studies showed that maximal and effective antinociceptive response to loperamide was observed in mice injected with apoE3-coated loperamide formulations that underwent a tail-flick test, suggesting transport across the BBB [49]. In other studies a comparative analysis of the antinociceptive effect of a hexapeptide dalargin (a synthetic equivalent of endogenous peptide enkephalin) was carried out by incorporating it into poly(butyl cyanoacrylate) nanoparticles and coating with apoAII, apoB, apoCII, apoE, or apoJ in the presence or absence of polysorbate 80 as a pre-coating material [49]. ApoE3 or apoB containing particles were found to be more effective in transporting these drugs across the brain capillary endothelial cells suggesting involvement of LDLr receptors.

### 3.5. Peptide Mimetics

In the last two decades, there has been intense focus on development of apo-based peptide mimetics as potential therapeutic agents for lowering cholesterol levels and treatment of cardiovascular diseases [10,135,136,137,138]. Critical aspects of the use of these 18–26 residue peptides are their high affinity for lipid binding and amphipathic helical nature, specifically class A motif, and features that essentially recapitulate an archetypical apolipoprotein. One of the most well studied of these mimetics is 4F (shown in bold), an 18 residue apoAI mimetic (DW**F**KA**F**YDKVAEK**F**KEA**F**) and its derivatives (tandem dimers of 4F linked by a Pro residue; substituting D-amino acids in place of L-amino acids to extend in vivo stability by decreasing protease susceptibility; changing degree of amphipathicity and net charge) that displayed potent cholesterol efflux and anti-inflammatory properties [139]. They bear 2–7 Phe residues on the nonpolar face of the helix and have the advantage of forming “tunable” nanodiscs, where the size can be modulated by changing the phospholipid/peptide ratios. Other possibilities include introducing Leu residues to tune hydrophobicity or peptides with variations in the number and position of Glu and Lys residues to tune amphipathicity [10,140] without significant alteration of the cholesterol efflux ability.

Based on the wealth of information available on these apoAI-based mimetics cholesterol efflux, other studies have developed their use for formation of nanodiscs for drug delivery. Saito and colleagues [141] have designed and developed an apoAI-based nanodisc scaffold peptide (NSP) that is bi-helical (two tandem helical peptides linked by a Pro residue). They bear the ability to form nanodiscs of varying diameters depending on the ratio of phospholipid and NSP and were more stable than nanodiscs formed from corresponding mono-helical peptides. Subsequently, a reverse variant of NSP (reverse sequence) without the N-terminal acetylation and C-terminal amidation, called NSPreverse (NSP_r_) was designed [142]. Many NSP_r_ enclosed proteins in the absence of lipids forming ‘peptidiscs’, which appear to be more effective in stabilizing various types of α-helical and β-barrel membrane-bound proteins. Other groups have employed commercially available apoAI mimetic peptides, 22A (PVLDLFRELLNELLEALKQKLK) and 5A (DWLKAFYDKVAEKLKEAFPDWAKAAYDKAAEKAKEA) peptides and egg sphingomyelin and/or POPC to form nanodiscs with prolonged circulation time in pharmacokinetic studies in rats [143], a desirable feature for drug delivery. Other peptides with powerful cholesterol effluxing capabilities, such as the apoE CT-domain-based peptides, have potential for drug delivery as well [144,145].

The ApoB-100-derived peptide bearing the LDLr binding segment (residues 3359–3367, RLTRKRGLK) with lipid-based [69,70] or peptide-based [67,68] anchors have been successfully employed to generate synthetic nano-LDL (Figure 2). For the lipid-based anchor, retinoic acid and cholesterol were conjugated to the N- and C-terminal ends, respectively, of the LDLr binding segment. For the peptide-based anchor, an 18 residue amphipathic α-helical peptide [146] was tagged to the N-terminal end of the LDLr binding segment (in italics) to generate a chimera (DWLKAFYDKVAEKLKEAF*RLTRKRGLK*LA) with the amphipathic segment serving as a lipid anchor. In both cases, the peptide was reconstituted with egg yolk phosphatidylcholine, triolein and cholesteryloleate to generate synthetic nano-LDL and successfully delivered cholesterol to lymphoma cells [69,70] or loaded drugs such as paclitaxel to GBM cells [67,68]. In a similar manner, the LDLr binding segment of apoE3 (residues 141–150) (sequence shown in Section 3.2) has been employed in a monomeric or tandem dimeric peptide form (residues 141–150/141–150) for complexation with sphingomyelin and cholesterol to form nanoliposomes with the aim of delivering a payload across the BBB [147]. The tandem dimeric peptide appeared to be more efficient than the monomeric form in mediating transcellular transport of tritiated curcumin following cellular uptake when tested in rat capillary cerebral endothelial cells, which points to endosomal/lysosomal escape of at least a portion of the added nanoliposomes. In an interesting variation, Sauer et al. [148] modified this sequence by appending either an amphipathic helical segment from apoE3 CT domain (residues 267-280, PLVEDMQRQWAGLV) or a sequence of hydrophobic residues (AWLALALALALKALALALALKK) that is known to adopt a transmembrane orientation in phospholipid bilayers at the C-terminal end or a lipid moiety (myristoyl, palmitoyl or dipalmitoyl group) at the N-terminal end. They noted that the dipalmitoyl group resulted in the most efficient internalization of lipoproteins in mouse brain capillary endothelial cells.

## 4. Mechanism of Assembly of the Lipid Platform of Apolipoprotein-Based Nanostructures 

Formulation of rHDL with cargo of interest takes advantage of two unique tendencies of apolipoproteins: (a) to spontaneously associate with lipid bilayer vesicles and promote self-assembly of nanodiscs at the gel to liquid–crystalline phase transition temperature (*T*_m_) of the phospholipid, and (b) to form nanodiscs with the assistance of detergents. The former process, termed vesicle solubilization, is a kinetically driven reaction that converts phospholipid bilayer vesicles (50–200 nm diameter) to nanodiscs (5–20 nm) and is determined by the *T*_m_ range and lipid composition [149,150,151,152]. The cargo of interest may be included during the initial stage of phospholipid preparation involving formation of thin films; while this is expected to alter the *T*_m_ and, therefore, the assembly process, optimal incorporation and assembly with apolipoproteins can be achieved by changing the incubation duration and temperature, and by co-sonicating the ternary mixture (lipid/cargo/protein). Although the exact mechanism of transformation is not known, it is proposed that the disordered state of the lipid presents packing defects that initiates binding at one end of the helix bundle in apolipoproteins [153,154,155], followed by penetration and helix bundle opening [156,157]. In addition, the low stability of the helix bundle structure in most apolipoproteins (apparent free energy of unfolding 1–4 kcal/mol, requiring <1.0 M guanidine HCl (GdnHCl) denaturant to induce 50% unfolding [GdnHCl]_1/2_) [156] contributes to its ease of opening with helix–helix interactions replaced by helix–lipid interactions. The NT domain of apoE3 is an exception since it bears a relatively stronger helix bundle (apparent free energy of unfolding of 8–12 kcal/mol; [GdnHCl]_1/2_ of ~2.5 M) [158]. Additional considerations when employing this method for apoE3 would include co-sonication of lipid, protein and cargo, lowering the pH of the buffer to induce a “molten globule” state to destabilize the tertiary interactions within the helix bundle structure, and/or inclusion of phospholipids with negatively charged head groups [159]. 

A second widely used approach involves a slight modification of the detergent-assisted process typically used to prepare rHDL [160]. Phospholipids with longer chain lengths (C16 and C18) bearing saturated and/or unsaturated fatty acyl chains can be employed. This process involves initial formation of binary, ternary, and quaternary mixed micellar complexes (for example, protein/detergent, lipid/cargo/detergent, lipid/protein/detergent/cargo, and variations thereof), which subsequently leads to formation of discoidal ternary complexes (lipid/protein/cargo) upon removal of detergent. The possible presence of trace amounts of detergent in the final rHDL needs to be considered as a potential source of toxicity for in vivo applications. In a variation of this method, spherical HDL can be reconstituted with a core containing either neutral lipids and/or lipophilic cargo. It is envisaged that the apolipoprotein component of these spherical nanostructures would be wrapped around the surface making more contact with the phospholipid head groups, and fewer van der Waal’s contacts with the fatty acyl chains than the discoidal complex (Figure 1).

Between the above two processes, a wide range of lipids has been utilized, a choice driven by the nature of the cargo, for example: synthetic phospholipids, including phosphatidylcholine, phosphatidylethanolamine, phosphatidylserine, phosphatidic acid, phosphatidylinositol 4,5-bisphosphate, and lecithin from egg yolk and soybean, cholesterol, cardiolipin, sphingolipids, diacylglycerol. The chain lengths vary from C13 to C18, and the degree of saturation include saturated and monounsaturated. By varying the initial lipid to protein ratio, nature of lipid (chain length, degree of saturation, inclusion of core-forming neutral lipids), apolipoprotein type, and size, a designer nanoparticle can be custom made to obtain a discoidal or spherical geometry, with a desired diameter (5–25 nm) suitable for the cargo.

Nanodiscs and spherical nanoparticles (Figure 1) are of appropriate dimensions and bear surface characteristics similar to that of endogenous nascent HDL. Overall, they present a unique and stable environment for biomolecule transport and delivery, with significant storage, lyophilization and freezing capabilities [75]. The presence of the protein envelope increases the stability and targeting specificity of the nanoparticles, despite their structural flexibility that allows them to undergo rapid association with, and dissociation from, lipoproteins. While several studies employ apoAI isolated from human plasma, others utilize recombinant forms of apoAI or apoE3 purified from *E. coli* or mammalian cell cultures. ApoE3 and its derivatives tend to yield nanodiscs of larger diameter (~20 nm) compared to apoAI (~10 nm), thereby allowing 3–4 times larger surface area for incorporation of biomolecules.

## 5. Synthesis of Apolipoprotein-Based Nanoparticles

**Figure 4:**
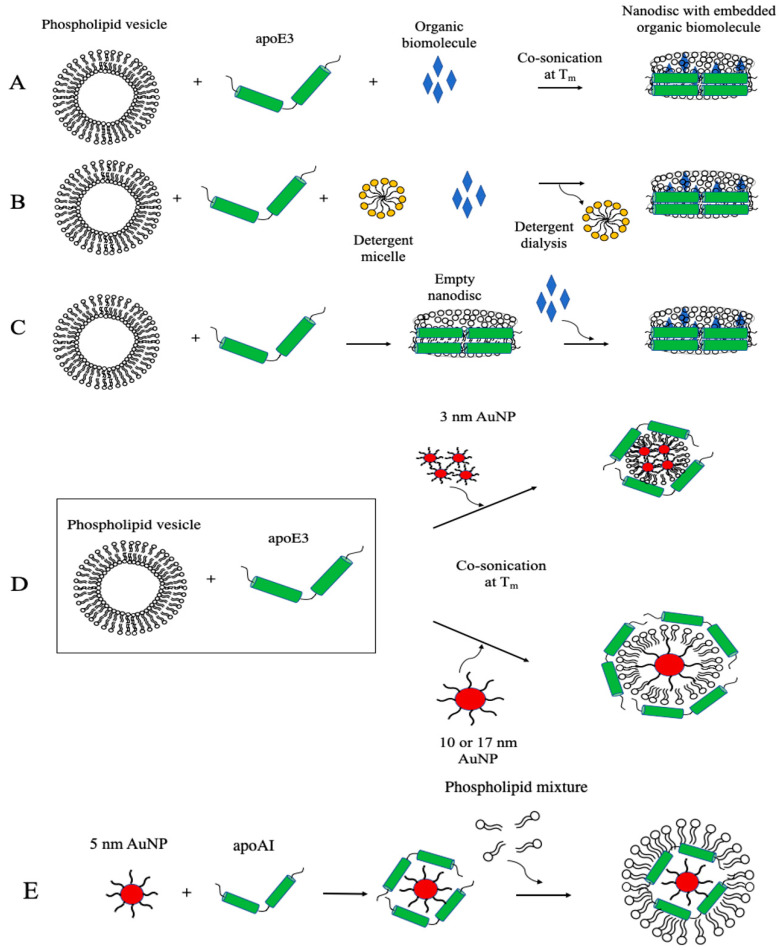
Incorporation of nonpolar molecules into apolipoprotein-based nanostructures. Nonpolar molecules’ incorporation into apolipoprotein-based nanostructures can be achieved by different approaches. (**A**) Co-sonication method; (**B**) detergent dialysis method; (**C**) incorporate hydrophobic molecules into pre-formed empty nanodiscs; (**D**) co-sonicate 3, 10 or 17 nm gold nanoparticles (AuNP) with phospholipid vesicles and apoE3; (**E**) build HDL-like lipoproteins around AuNP as a core template with apoAI and phospholipids.

Figure 4 illustrates standard approaches employed to incorporate lipophilic or amphipathic organic and inorganic molecules into apolipoprotein-based nanostructures. The spontaneous nanodisc formation approach involves initial co-solubilization of lipids and cargo in a suitable solvent mixture such as chloroform/methanol, drying under nitrogen and further removal of solvent to form a thin film. This is followed by hydration of the lipid film to form multilamellar or large unilamellar vesicles, addition of apolipoproteins in buffer (in the required lipid: protein: cargo mass ratio), and sonication in a bath sonicator at the T_m_ of the lipid (for example at 24 °C for 1 h for DMPC) to yield a clear solution of nanodiscs (Figure 4A). The clear solution is subjected to potassium bromide density gradient ultracentrifugation [39,63,161] to separate protein-free vesicles from rHDL/cargo and lipid-free protein and free cargo. Alternately, size exclusion chromatography may be used for a gentler separation of rHDL with embedded cargo. The detergent-assisted method involves a similar initial step as above to form lipid or lipid/cargo thin film. The hydration step, however, includes a detergent such as sodium cholate [149] or deoxycholate and apolipoprotein solution with detergent-to-lipid molar ratio ranging from 1:1 or 4:1 (Figure 4B). The detergent is removed by extensive dialysis or by Bio-Bead absorption or passage through size-exclusion columns [160,162,163], and the resulting nanodiscs isolated by density gradient ultracentrifugation or by size-exclusion chromatography as described above. Formulation of lipoproteins by this method should consider the presence of trace amounts of detergent as a potential source of cytotoxicity. Other studies demonstrate direct incorporation of lipophilic molecules into preformed empty nanodiscs, Figure 4C. In this case, it is important to restrict the amount of solvent in which the cargo is dissolved to <5% v/v, to maintain the integrity of the bilayer complex. For rapid, large-scale single-step preparation of apoAI-containing HDL of a defined, reproducible size range, Kim et al. [164] applied microfluidic technology with a flexible platform to obtain HDL-mimicking nanomaterials (μHDL) that can be loaded with chemically diverse cargo.

Nanoprecipitation is frequently employed to synthesize rHDL with core–shell components with gold nanoparticles (AuNP). In this approach, the hydrophobic AuNP are typically dissolved in chloroform/methanol solution along with lipids, followed by dropwise addition of the mixture into hot deionized water with stirring to allow formation of core–shell nanoparticles as the organic solvent evaporates. Following overnight incubation with the apolipoprotein, the mixture is subjected to density gradient ultracentrifugation for the purification of AuNP-HDL [165].

In a variation of this method, DMPC thin film, recombinant His-tag apoE3 and 3, 10 or 17 nm citrate-capped AuNP (5:2:3 w/w ratio) were co-sonicated in buffer and incubated for 16 h at 24 °C [64] (Figure 4D). The mixture was centrifuged at low speed, the bottom fraction washed several times with buffer, and His-tag/apoE3-associated AuNP captured by immobilized metal affinity chromatography. This procedure appeared to yield nanoparticles that had several 3 nm AuNP/particles or a single 10 or 17 nm AuNP/particle ensconced by a phospholipid monolayer and apoE3 (discussed further below). This approach best recapitulates the native interaction of apoE3 and phospholipids, and proper structural conformation of apoE3 that allows it to bind to LDLr. Since larger, metallic hydrophobic nanoparticles are more difficult to synthesize due to the absence of surface charge to shield against the electrostatic attraction of the metal cores, a phase transfer method using a co-solvent and surfactant were needed to protect the aqueous AuNP during the transfer process. This allows adsorption of alkanethiols that can form a monolayer onto the nanoparticle surface. The size of the metal core can be controlled to allow generation of lipoproteins of varying size. 

On the other hand, Thaxton and colleagues [77,166] modified the lysines on apoAI with Traut’s reagent to introduce sulfhydryl groups in an effort to anchor the protein onto the surface of the core AuNP. They employed 5 nm citrate-stabilized AuNP as a template to allow adsorption of apoAI by overnight incubation at room temperature followed by treatment with a 1:1 mixture of DPPC and 1,2-dipalmitoyl-*sn*-glycero-3-phosphoethanolamine (DPPE) derivative; the organic solvent was removed by heating to 65 °C, followed by repeated low-speed centrifugation of the mixture. Their proposed templated HDL nanoparticles [77] appeared to be composed of a core of AuNP surrounded by a mantle-like apoAI and a crust-like phospholipid (Figure 4E). Transmission electron microscopy (TEM) images showed uniform “rings” of lipids around the nanocrystal core via negative staining, indicating a core-shell morphology. Although the exact orientation of apoAI on the surface of these nanocrystals remains to be investigated, Luthi et al. [167] hypothesized a snorkel model of the amphipathic helices based on the relatively consistent size upon apoAI addition to the Au-phospholipid surface as estimated by TEM. The authors attributed the insignificant size difference to the apoAI nestling in the lipid bilayer, with the nonpolar regions of the helices interacting with the lipid tails and polar regions facing the lipid head groups [166,168]. Taken together, the methodology used to obtain apolipoprotein-based nanoparticles (summarized in Table 2) will be determined by the nature of the payload, the lipid platform, protein scaffold, the target, and potential application.

## 6. Organic Biomolecules Payload in Apolipoprotein-Based Nanostructures

**Figure 5:**
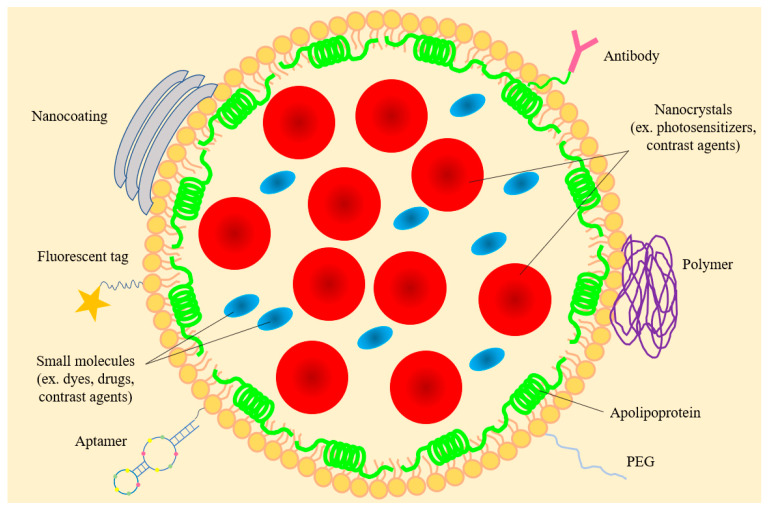
Applications of rHDLs as transport vehicles. rHDLs can serve as nanovehicles for transporting hydrophobic, amphipathic, and hydrophilic substances. The surface of rHDLs can be conjugated with antibodies or aptamers/nucleic acids for enhancing targeted delivery or modified with coating materials and polymers for extending circulation time inside the body.

In general, HDLs offer three chemically distinct environments to accommodate biomolecules via noncovalent interactions: the core, shell (or mantle), and corona (or crust), roughly corresponding to the interior, the interface, and the periphery of the lipoproteins. Lipidomic and proteomic analysis reveals that plasma HDL is a heterogenous mixture of lipoproteins [38] with >80 different proteins, >200 lipid species, and at least 10 microRNAs. The lipids in HDL include major lipids (phospholipids, triglycerides, cholesterol, CE, sphingolipids), minor lipids, such as mono-, diacylglycerides, free fatty acids, and a host of other lipophilic biomolecules [173] such as oxidized lipids, hormones and steroids, eicosanoids, lipid-soluble vitamins, coenzyme Q, antioxidants, quinones, and pigments. Borrowing transportation strategies from the naturally occurring HDL, investigators have been successful in reconstituting synthetic HDL (defined as density 1.06–1.21 g/mL) with a broad range of chemically and functionally diverse organic biomolecules, depicted in Figure 5, and broadly classified here as: antioxidant, anti-inflammatory and anti-atherogenic agents, nucleic acid agents, and anti-cancer and antimicrobial agents.

### 6.1. Antioxidant, Anti-Inflammatory, and Anti-Atherogenic Agents 

Poloni et al. [174] carried out a systematic investigation comparing the binding of 41 plant polyphenols, which bear potent antioxidant properties, to serum albumin and LDL isolated from pigs. By monitoring quenching of intrinsic fluorescence of albumin and apoB-100 on LDL following addition of polyphenols, they reported that the calculated quenching constant and binding were in general higher for albumin compared to LDL. Nevertheless, the presence of even one molecule of polyphenol/LDL (which can accommodate at least ~10) may offer sufficient antioxidative protection, and the ability to be transported to intracellular sites via the LDLr.

**Figure 6:**
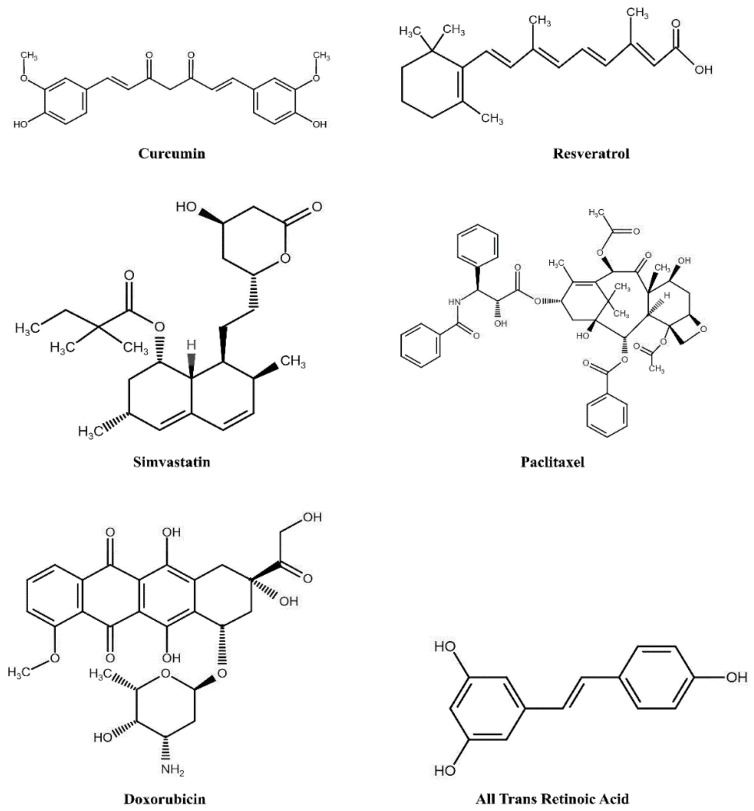
Chemical structures of select hydrophobic agents that have been incorporated into lipoproteins.

Analogous to LDL, rHDL offers a similar microenvironment for accommodating polyphenols such as diferuloylmethane, (1,7-bis(4-hydroxy-3-methoxyphenyl)-1,6-heptadiene-3,5-dione, commonly referred to as curcumin) (Figure 6). Curcumin is a phytochemical derived from the rhizome of *Curcuma* species, Zingiberaceae. It bears antioxidant and anti-inflammatory properties [175] and shows potential for cardioprotective effects by its ability to raise plasma HDL levels and functionality [176]. Its lipophilic nature allows it to partition efficiently into the hydrophobic milieu of the phospholipid bilayer of nanodiscs with apoE3-NT (~17 nm diameter) as evidenced by fluorescence quenching assays, without compromising the LDLr binding activity of apoE3 [18]. Others noted that rHDL bearing apoE3 was more potent in delivering curcumin to GBM cells and showed a greater decrease in cell viability (~25%) compared to free curcumin or rHDL bearing apoAI (55%) [39,161,169] which supports the proapoptotic and growth inhibitory properties of curcumin. Nanodiscs generated with apoAI, curcumin, and DMPC was reported to be larger in diameter (~50 nm) compared to that with apoAI and DMPC (5–15 nm) and appeared to inhibit cell growth in hepatoma cells and induce greater apoptotic response (~70%) in mantle cell lymphoma more efficiently than free curcumin (~20%) [161,169]. These reports indicate that rHDL (with apoE3 and its derivatives or apoAI) serves to overcome the poor water solubility of curcumin; more studies are needed to assess the biological half-life of rHDL/curcumin and its bioavailability in a non-metabolized form and to compare its ability to combat intra- and extracellular oxidative stress. In other studies, the anti-amyloidogenic activity of curcumin was assessed in AD transgenic mice models [177,178,179]. They noted its ability to bind Aβ and inhibit its oligomerization and fibrillization, and to reduce oxidative damage and amyloid pathology suggesting that rHDL-mediated delivery of curcumin across the BBB may further enhance its potential to treat amyloid pathology associated with neurodegenerative diseases.

**Figure 7:**
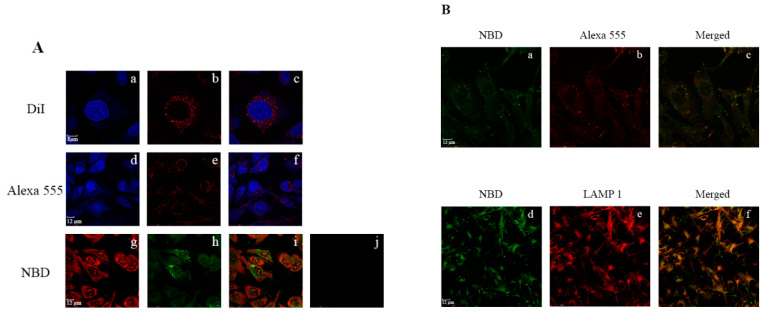
Lysosomal targeting of rHDL/res/NBD by apoE3 in glioblastoma cells. (**A**) Uptake of individual components of rHDL/res was monitored by direct or indirect fluorescence: lipid (**a**–**c**), apoE3 (**d**–**f***)* and resveratrol (**g**–**i**). Following exposure to rHDL/res/DiI at 37 °C for 3 h (**a**–**c**), the cells were visualized under a confocal laser scanning microscope: (**a**) DAPI; (**b**) DiI; (**c**) merge of (**a**) and (**b**). Following exposure to rHDL/res under the same conditions (**d**–**f**), the cells were visualized by: (**d**) DAPI; (**e**) apoE3 monoclonal antibody, 1D7, and Alexa555-conjugated secondary antibody; (**f**) merge of (**d**) and (**e**). Following exposure to rHDL/res/NBD (5 μg) as described above (**g**–**i**), the cells were visualized by: (**g**) propidium iodide; (**h**) resveratrol conjugated to 4-chloro-7-nitrobenz-2-oxa-1,3-diazole (res/NBD); (**i**) merge of (**g**) and (**h**). Panel (j) shows that uptake of res/NBD is minimal in the absence of rHDL. (**B)** Co-localization of res/NBD with apoE3, or with LAMP1 in late endosomal/lysosomal vesicles following cellular uptake of rHDL/res/NBD. Following exposure to rHDL/res/NBD, the cells were visualized by fluorescence associated with: (**a**) NBD to detect res, (**b**) Alexa555-conjugated secondary antibody to detect apoE3; (**c**) merge of (**a**) and (**b**); (**d**) NBD to detect res; (**e**) Alexa 594-conjugated secondary antibody to detect LAMP1; (**f**) merge of (**d**) and (**e**). Reproduced with permission from [63]. Copyright Public Library of Science, 2015.

Resveratrol (3,5,4′-trihydroxystilbene) (Figure 6) is another plant derived polyphenol that bears anti-atherogenic [180,181,182] and antioxidant [181,183,184] activities. It is found in both cis- and trans-isomeric forms in grape skins, peanuts, and some berries, and better known as one of the components of red wine. At the biochemical and molecular level, the trans-isomer is more active than the *cis* form and appears to mediate its role through activation of Sirt1 [184,185], a NAD^+^-dependent protein deacetylase, which regulates a wide variety of signaling pathways. Oral consumption of resveratrol leads to low plasma levels of the active form, since the liver converts it to the relatively polar glucuronidated and sulfated derivatives [186,187,188], which are not as effective as their unmodified form as an antioxidant. Alternative approaches have been formulated to incorporate resveratrol and to increase its aqueous solubility and bioavailability, including polymer- and lipid-based nanoparticles and nanocapsules, or cyclodextrin-based nanosponges [189]. The LC-MS analysis of plasma lipoproteins of subjects who consumed a limited amount of red wine indicated the presence of resveratrol and its glucuronide derivatives in LDL [190]. Two independent studies investigated the effect of incorporation of resveratrol in apoE3-based nanoparticles [63,171]. In one, rHDL containing apoE3-NT and DMPC was formulated with ~4–6 molecules of trans-resveratrol per nanodisc (~20 nm diameter). The presence of resveratrol did not affect the presentation of the multivalent receptor binding sites or the LDLr binding activity of apoE3-NT. Fluorescence imaging of GBM cells treated with apoE3-NT bearing rHDL loaded with resveratrol conjugated to 4-chloro-7-nitrobenz-2-oxa-1,3-diazole (res/NBD) revealed significant perinuclear punctate distribution of fluorescence in endosomal/lysosomal vesicles (Figure 7). In contrast, treatment with res/NBD in DMSO under identical conditions did not show any cellular fluorescence, demonstrating successful intracellular delivery of resveratrol via apoE3-based nanodiscs. In the other study, researchers used apoE3 derivatized with biotin and bound to avidin that was conjugated to solid lipid nanoparticles composed of cetyl palmitate and polysorbate 80 [171] to deliver resveratrol across an in vitro model of the BBB using human cerebral microvascular endothelial cells. They noted a 2-fold enhancement of resveratrol transport across the monolayer when it was functionalized with apoE3 compared to its non-functionalized state, a process likely mediated by member(s) of the LDLr receptor family of proteins. Taken together with observations that resveratrol promotes clearance of Aβ peptide [191], apoE-mediated delivery of resveratrol across the BBB offers potential to decrease amyloid pathology in AD. 

Simvastatin, a lipophilic statin (Figure 6) that inhibits 3-hydroxy-3-methylglutaryl coenzyme A reductase (HMG-CoA reductase), a key enzyme in cellular cholesterol biosynthesis pathway, is prescribed as an oral LDL cholesterol lowering drug. High levels of plasma LDL cholesterol is an established risk factor for developing cardiovascular disease. Simvastatin also upregulates LDLr expression in the liver. In macrophages, HMG-CoA reductase inhibition can elicit anti-inflammatory response and thereby modulate atherosclerotic plaque formation. Duivenvoorden et al. [170] packaged simvastatin in rHDL containing apoAI, DMPC, 1-myristoyl-2-hydroxy-sn-glycero-phosphocholine (a lyso PC) (s-[rHDL]) and tested the therapeutic potential of s-[rHDL] as an anti-inflammatory agent. They used a low dose of apoAI in an effort to uncouple the cholesterol lowering and other therapeutic effect of rHDL itself (as described earlier) from that of statin’s. A decreased generation of inflammatory cytokines and macrophage viability was noted, with very little toxic effect on liver, kidney, or myocytes when s-rHDL was administered at 60 mg.kg^−1^ simvastatin, a dose that typically induces liver and muscle toxicity if simvastatin is administered as such. When injected into apoE-knockout mice, a model system for atherosclerosis, they noted that s-rHDL accumulated in atherosclerotic lesions, decreased vessel wall thickness and total plaque area, and inhibited plaque inflammation progression compared to rHDL or placebo group.

Overall, employing rHDL loaded with statins or other synthetic or naturally occurring antioxidant, anti-inflammatory biomolecules (for example, tanshinone, a class of abietane diterpene molecules) or a combination approach shows promise for treating atherosclerosis [192,193].

### 6.2. Nucleic Acid Agents

The last two decades have witnessed the emergence of the use of small interfering RNAs (siRNA) as potent ways to silence or “switch off” expression of specific proteins by virtue of their ability to selectively bind mRNAs. Some major impediments to using siRNAs to treat human diseases in clinical practice include the lack of targeting and effective delivery vehicles. The use of rHDL addresses the latter by offering a relatively protected microenvironment, with the apolipoprotein component bearing potential to enhance targeting. Aptamers or siRNAs are small double stranded nucleotides whose polar and anionic nature pose challenges to enter cells.

Shahzad et al. [46] used rHDL to test the efficacy of two different siRNAs with the goal of silencing signal transducer and activator of transcription 3 (STAT3) and focal adhesion kinase (FAK). STAT3 is involved in normal chemical signaling pathways in cell proliferation, migration, and apoptosis and is activated in tumors, and therefore has to be selectively silenced; FAK is essential for tumor metastasis. These authors demonstrated efficient incorporation of STAT3 and FAK siRNA into synthetic HDL reconstituted with cholesterol, cholesteryl oleate, egg yolk phosphatidylcholine and apoAI by the cholate dialysis method. They noted significant reduction in STAT3 and FAK expression levels in vitro and provided proof of concept for its use in therapy by testing the efficacy of rHDL bearing siRNA in mouse models of metastatic of ovarian and colorectal cancer. A significant reduction in tumor growth was noted in both cases (particularly with combination therapy with docetaxel or oxaliplatin) with the effects noted in terms of lowered tumor cell proliferation, and angiogenesis and cell survival, with the entry likely mediated via SR-B1. The efficiency of incorporation of siRNA into rHDL nanoparticles was enhanced by neutralizing the negative charge on the siRNA with 40-mer poly-lysine. Others have used cationic lipids to improve incorporation into rHDL containing apoAI [194]. In addition, apoE3’s ability to bind lipoprotein receptors has further been harnessed to improve cellular uptake for effective RNA interference by non-covalent interactions of the protein coat around lipid nanoparticles containing siRNA, cholesterol and PEGylated 1,2-dimyristoyl-sn-glycerol (DMPG) and ionizable amino lipid YSK05 (1-methyl-4,4-bis(((9Z,12Z)-octadeca-9,12-dien-1-yl)oxy)piperidine) [195,196]. The resulting complex called apoE-YSK-multifunctional envelope-type nano device (apoE-YSK-MEND) was significantly more efficient in silencing β-site amyloid precursor protein cleaving enzyme 1 (BACE1) (from which Aβ peptide is generated) in Neuro-2a cells compared to YSK-MEND alone, indicative of effective cellular uptake in the presence of apoE3. 

In a similar approach, synthetic HDL-like particles were constructed around a core of head group modified DPPE that was chemically activated to enable conjugation of small oligonucleotides of varying lengths, and coated with DPPC and apoAI [197]. These lipid conjugated HDL-like particles elicited strong cholesterol efflux ability (from J774 macrophages) and delivery ability (to hepatocytes (HepG2 cells)). In an interesting variation, a synthetic HDL composed of a 5nm-AuNP core surrounded by apoAI and a crust-like phospholipid with cholesteryl-DNA was engineered [168]. Antisense sequence to miR-210 was used to interact with, and relieve repression of, miR-210 targets such as E2F3a and to induce STAT1, another member of the STAT family of transcription factors. A significant reduction in miR-210 and target protein levels was noted in PC-3 prostate cancer cells, with minimal non-specific interactions and cell toxicity. Cholesterol has also been conjugated to the 3′ end of chemically modified siRNA with partial phosphorothioate backbone and 2′-O-methyl sugar modification to target apoB-100 (cholesterol-siRNA-apoB-100) [198] (apoB-100 and lipoproteins with apoB-100 are considered atherogenic) and co-administered with HDL or LDL that were isolated from plasma to mice [199]. This resulted in improved silencing of liver apoB-100 mRNA compared to cholesterol-siRNA-apoB-100 alone. While HDL directed the delivery of siRNA to tissues rich in lipid metabolism and lowered apoB-100 transcript levels (liver, gut, kidney and steroidogenic organs), LDL directed it predominantly to the liver, suggesting the involvement of SR-B1 and LDLr, respectively. Conjugation of siRNA with other hydrophobic molecules such as long chain saturated fatty acids (>C18) but not medium chain (C12 to C16) was effective in reducing apoB-100 levels in the liver.

In subsequent developments, incubation of cholesterol-siRNA-apoB-100 with POPC/apoAI or DMPC/apoE3 rHDL was reported to result in >90% incorporation, a process that appears to be independent of the siRNA sequence [97]. It was found that DMPC/apoE3 rHDL bearing cholesterol-siRNA-apoB-100 was more efficient and effective compared to POPC/apoAI in terms of reducing apoB mRNA in liver, and apoB-100 and LDL cholesterol levels in plasma. Further, a 1:1 loading of siRNA to lipoprotein complex appeared to be more effective than a 3:1 complex, indicative of possible charge–charge repulsion between multiple siRNA molecules on the surface of the discoidal particle and the lipoprotein receptor ligand binding domain which bears an abundance of negatively charged residues. This platform was adapted also for the delivery of anti-vascular endothelial growth factor receptor 2 (VEGFR2) oligonucleotides to suppress angiogenesis [200], to knockdown VEGFR expression in human umbilical vein endothelial cells and to promote cell survival. Overall for gene-based therapy with siRNA and other nucleic acids such as adenoviral DNA [201] delivery via the HDL platform holds promise and potential [202] especially for cancer treatment. Further optimization is needed with focus on targeting capability, decreasing enzymatic degradation and identifying effective endosomal/lysosomal escape, dosage and half-life.

### 6.3. Anti-Cancer and Antimicrobial Therapeutics

While the fields of cancer and antimicrobial therapy have made significant strides in developing potent therapeutic agents, their effectiveness has been hampered by significant biological and physio-chemical barriers, such as poor aqueous solubility, transmembrane transport, targeting and bioavailability of active forms of the drugs. Many nanotherapeutic applications have addressed these barriers to a large extent, but there is still an unmet need to fine tune the delivery strategy. Lipoprotein researchers have taken advantage of the HDL structure, chemistry, and biology to address many of the above issues to cancer and antimicrobial treatment.

Paclitaxel (PTX), a tetracyclic diterpenoid as shown in Figure 6 (commonly known as Taxol in free form or Abraxane when complexed to albumin), is a chemotherapeutic agent, that is currently being administered to treat several cancer types. Its mode of action involves disrupting microtubule function during cell division. Incorporation of PTX in apoAI-containing rHDL has shown to increase its stability and cytotoxicity, and enhance its anticancer properties compared to free PTX [40,41], with reports of up to ~100 PTX molecules incorporated into 12 nm sized spherical rHDL nanoparticles [42]. Its cytotoxicity was evaluated in ovarian, prostate and breast cancer cell lines and found to be more effective with IC_50_ values 5–20 fold lower than those for free PTX. It was noted that rHDL/PTX treated mice models had significantly lower weight loss than in those of PTX-treated group. As expected, uptake of rHDL/PTX correlated with SR-B1 expression as inferred from competition studies in prostate cancer cells, wherein ~80% of the total incorporated PTX was taken up [41]. In an alternate approach, rHDL containing PTX has been prepared with POPC, cholesterol esterified with succinate and modified apoAI with the intent to decrease loss of PTX due to the particle remodulation by enzymes such as lecithin cholesterol acyl transferase [203]. While these particles showed promise in terms of better efficacy in vitro and in vivo compared to rHDL-bearing PTX and unmodified cholesterol [204], it does not preclude a dynamic interexchange of lipids, apoAI, and PTX with plasma lipoproteins, an aspect that deserves more attention in future studies.

In contrast to the issue of drug leakage from rHDL, controlled drug release was studied with doxorubicin (Dox) hydrochloride, a hydrophilic anthracycline-based drug (Figure 6) that acts by inhibiting topoisomerase 2 [43]. Doxorubicin was incorporated into rHDL prepared from apoAI and egg phospholipids (rHDL-Dox) and its release compared to that of free Dox and Dox incorporated into liposomes. Doxorubicin incorporated in rHDL showed a sustained release in vitro in release studies with ~45% released 36 h after dialysis compared to ~90% and 55% for free Dox and Dox in liposomes, respectively. Higher accumulation of rHDL-Dox was observed in cells expressing SR-B1, with rHDL mediated delivery being more efficient in reducing tumor growth compared to liposome delivered Dox. Derivatives of Dox such as valrubicin, currently approved for intravesical treatment of bladder cancer (direct injection of liquid into a patient’s bladder), elicit enhanced fluorescence characteristics upon incorporation into a hydrophobic environment, such as the core of apoAI containing rHDL, showing additional potential for theranostic or imaging applications [47,48].

Other combinations of therapeutic (anticancer, anti-microbial) agents and apoAI variants have been incorporated into rHDL and targeted to SR-B1 with significant success; some examples include: 10-hydroxycamptothecin, a topoisomerase 1 inhibitor with apoAI-Milano [44]; withalongolide A 4,19,27-triacetate, an inducer of oxidative stress response and inhibitor of HSP90, a molecular chaperone, without or with 5-fluorouracil, and with apoAI mimetic peptide 22A [45]; all-trans retinoic acid (Figure 6), a retinoid ligand that binds retinoic acid/retinoic X receptors and serves as a transcription factor for proteins involved in cell differentiation [205,206,207]; everolimus an inhibitor of mammalian target of rapamycin [208]; Sorafenib, a multi-target kinase inhibitor in combination with antimiRNA21 [209]; pH-responsive cell penetrating peptide (RRRRRRHHHH) to improve penetrating potential of apoAI-containing nanodiscs to deliver an antitumor agent gambogic acid [210]; antifungal agents, such as amphotericin B, a polyene antibiotic [211] and rapamycin [212]; and lactosylated rHDL to deliver antiviral drugs to liver [213,214]. Overall, as far as cancer therapeutics are concerned, whereas SR-B1 would be the first point of entry at the plasma membrane of cells, some drugs, such as topoisomerase inhibitors and transcription factors, would have to cross a second barrier, the nuclear membrane.

## 7. Metal Core in Apolipoprotein- Based Nanostructures for Imaging, Diagnostics, and Therapeutics

### 7.1. Metal Core in Nanolipoproteins as Diagnostic Tool

This section focuses on the use of HDL as a model system for imaging and diagnostic purposes by incorporating iron oxides (FeO) for magnetic resonance imaging (MRI), AuNP for CT and TEM, and quantum dot (QD) nanocrystals for fluorescence imaging via confocal microscopy. The core, shell and corona may be modified to generate multifunctional platforms suitable as theranostic agents [215], by reconstituting HDL with lipids, apoAI, and various inorganic nanocrystals (e.g., gold, FeO, and QDs) for imaging atherosclerosis. Notably, compared to PEGylated nanocrystals, the HDL counterparts demonstrated superior cell uptake in macrophages via SR-B1 and ABCG1. In a refinement of this approach designed to facilitate spectral (or multicolor) CT imaging of atherosclerotic plaques in apoE-null mice, a combination of AuNP-HDL (reconstituted with rhodamine–lipids) and iodine-based contrast agents were administered in tandem, the former via i.v. injection, followed by the latter a day later to visualize blood vessels. AuNP-HDL accumulation was detected in macrophages by TEM and confocal microscopy of aortic sections. The contrast agent and calcium phosphate rich tissue were detected allowing simultaneous visualization of vessels and bones in a single scan [165]. This innovative combination of AuNP-HDL and spectral CT has other potential applications in detecting stenosis and thrombosis to treat cardiovascular disease and stroke.

Iron oxide nanocrystal core in rHDL also serves as a good image contrast agent, particularly for MRI, optical imaging and TEM allowing visualization at the tissue, cellular and sub-cellular levels. Further, FeO-HDL with a fluorophore embedded in the phospholipid layer was found to be biocompatible, mimicked the activities of endogenous HDL, elicited cholesterol efflux capabilities, and were internalized by hepatocytes and macrophages wherein they localized in endosomes [216]. Similarly, other paramagnetic contrast agents, such as gadolinium (Gd), have been reconstituted into apoAI or apoAI-mimetic peptide with phospholipids and fluorophores for visualization in aortic plaques when injected in mice [217,218]. 

Compared to Au or FeO, fewer studies focus on reconstituting QDs for biomedical applications, likely due to the fact of their toxicity [219]. Nevertheless, their intrinsic fluorescent properties make QDs excellent agents for optical studies. A FRET-based platform that studies lipid exchange using lipoprotein-reconstituted QDs was developed [220], which consisted of cadmium selenide (CdSe) core–cadmium sulfide (CdS) shell-zinc sulfide (ZnS) shell organization. By incorporating near-infrared responsive Cy5.5 dyes in the lipid coating that could be excited from the photon emitted by the QD donor core, FRET was monitored between HDL-QD and Cy5.5, thereby providing the ability to monitor lipid exchange [221]. Since human tissue is permeable to near infra-red (NIR) light with minimal damage, CdSe-QD based HDL bear potential for in vivo imaging.

### 7.2. Metal Core in Nanolipoproteins as Therapeutic Agents

**Figure 8:**
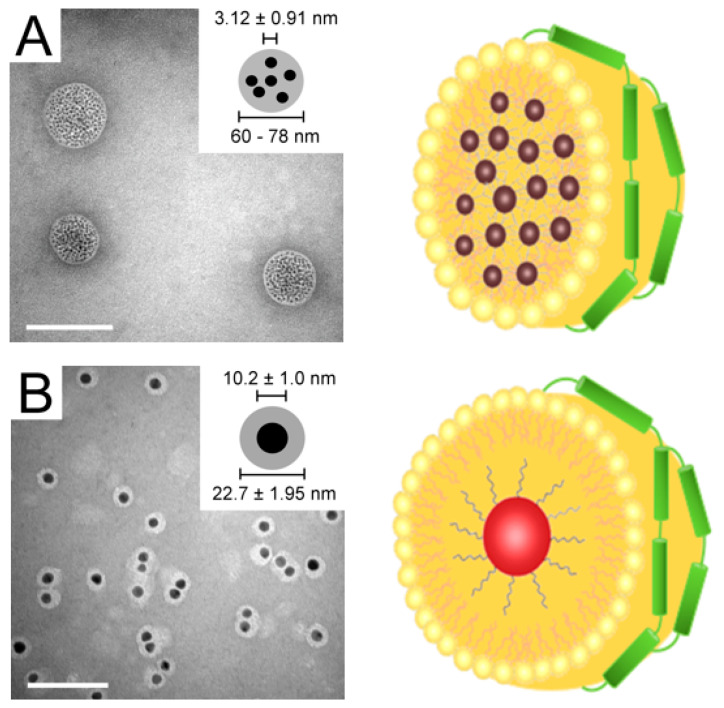
Nanolipoproteins reconstituted with 3 nm and 10 nm AuNP. TEM image (**Left**) and schematic representation (**Right**) of 3 nm (**A**) and 10 nm (**B**) AuNP incorporated into rHDL/apoE3. The scale bar represents 100 nm in TEM images. TEM image of rHDL-AuNP prepared with 3 nm AuNP (**A**) revealed 60–80 nm spheroid structures with several AuNP (Inset, **A**), while that prepared with 10 nm AuNP revealed ~23 nm spherical structures with a single AuNP (Inset, **B**). The light area around the 10 nm AuNP likely represents the lipoprotein shell. Reproduced with permission from [64]. Copyright Dove Medical Press, 2017.

In addition to imaging, AuNP have also attracted attention due to their photothermal property, with potential application in photothermal therapy. Photothermal therapy is a form of hyperthermia used in cancer theranostics [222] to release heat upon absorbing light in the NIR window (650–950 nm), a feature that is appealing for medical applications, especially for deep tissue tumors. The relatively low absorption of AuNP in the NIR window can be improved by causing them to cluster or aggregate. Aggregation of AuNP may also be induced by lowered pH environment such as that in the late endosomes/lysosomal vesicles as noted upon incubation of rHDL/apoE3 bearing 3, 10 or 17 nm AuNP with GBM cells (Figure 8) [64]. The embedded AuNP did not affect the LDLr binding capability of rHDL/apoE3. The naturally occurring intracellular aggregation of AuNP is an appealing feature to apply photothermal therapeutic strategy in chemo-resistant cancer cells or for disassembly of amyloid aggregates. A similar strategy using pH-triggered aggregation was also demonstrated by Hainfeld and colleagues [223]. By decorating the AuNP surface with ligands rich in carboxyl groups, it was shown that these particles readily aggregate under endosomal and lysosomal pH, in which the protonated carboxyl groups are unable to sustain the interparticle charge repulsion, thus leading to particle aggregation. Further, inclusion of a curcumin fluorescent motif in HDL/apoE3 reconstituted with AuNP caused disassembly of aggregated Aβ(1-42) in vitro [172]. By monitoring the fluorescence of curcumin, the fluorescence intensity was correlated to the levels of Aβ aggregation, with an increase in fluorescence indicating its partitioning into Aβ aggregates. The presence of AuNP in rHDL platform has the potential to trigger photothermal irradiation induced disaggregation of Aβ assembly.

Others showed that the AuNP-HDL possess the ability to efflux cholesterol from human and murine macrophages [167] with the size of the template AuNP and the leaflet organization of the surrounding phospholipids impacting the degree of cholesterol efflux. This is supported by the observation that HDL reconstituted with 6 nm AuNP surrounded by a bilayer of phospholipids showed the highest degree of cholesterol efflux compared to that with 8 nm AuNP and a monolayer. The difference was attributed to the curvature and packing of phospholipids on the surface of AuNP, rather than the surface area of the particles. Importantly, this synthetic construct showed a near 2 fold greater cholesterol efflux compared to endogenous human HDL. This capacity to accept effluxed cholesterol not only implies its therapeutic potential for treating atherosclerosis, but also for cancer treatment by perturbing cholesterol homeostasis in tumor cells.

Natural HDL, when bound to SR-B1, releases CE while shrinking in size. The synthetic HDL-AuNP on the other hand, has a rigid metallic core that does not get consumed in the subsequent process upon receptor binding. Utilizing this advantage to deplete cancer cells of cholesterol, leukemic cells derived from patients with chronic lymphoid leukemia were treated with HDL-AuNP [224]. Flow cytometry analysis of peripheral blood mononuclear cells showed no apparent toxicity, due to the absence of SR-B1 expression in these cells. When treated with 30 and 100 nM HDL-AuNP a greater percentage of cells was observed to undergo apoptosis. Closer examination revealed a dose-dependent cytotoxic effect in the lymphoid leukemia cells, while the healthy T cells and B cells, which did not express SR-B1, were spared.

Therapeutic interventions that seek to suppress myeloid-derived suppressor cells (MDSCs), a heterogenous population of myeloid precursor cells that exert a negative immune response [225] have gained interest in recent years. Recently, it was demonstrated that HDL-AuNP can further enhance T cell activity by reducing the activity of MDSC [226]. It was noted that MDSC, like lymphoma, also expressed SR-B1 and that HDL-AuNP inhibit MDSC activity and reduce tumor growth and metastatic tumor burden primarily by acting via SR-B1. HDL-AuNP did not induce cytotoxicity in the healthy cells. Importantly, HDL-AuNP treatment increased the proliferation of CD4^+^ and CD8^+^ T cells, which are important for adaptive immunity against tumors but are often suppressed by MDSC. To further understand the mechanistic details, the authors performed RT-PCR and showed a decrease in the expression of MDSC biomarkers such as *S100A9* and *ARG1*. Knockout studies of SR-B1 showed that the anti-tumor effect was exerted through the SR-B1 axis. This translates to a significant increase in survival rate in the mice, as pretreatment with three doses of HDL-AuNP decreased lung melanoma metastasis. 

## 8. Summary and Future Perspectives

The last four decades have witnessed a deeper understanding of apolipoprotein structure and function in lipid-free and lipid-associated states that has led to the appreciation and parallel development of synthetic lipoproteins for drug delivery and imaging. The merits associated with nanolipoproteins include tunability in terms of size, modularity in terms of being able to reconstitute ternary or quaternary complexes, and tailorability in terms of ease of synthesizing custom made and designer platforms with different types of lipids and apolipoproteins. The latter addresses possibility of developing targeted “zip-code”-based delivery of therapeutics using chimeric apolipoproteins [227] currently a challenge in this field especially in cancer therapy. At the tissue/cellular level, the BBB still remains a formidable hurdle despite significant efforts to exploit receptors such as LRP [228] and SR-B1 as initial points of entry to precede transcytosis across the endothelial layer at the neurovascular junction. While modification of apolipoproteins may expand our toolbox in terms of targeting, the ubiquitous nature of the receptors still poses a challenge, demanding further scrutiny in this area. There is scope for fine-tuning at the subcellular level, for treating mitochondrial or lysosomal storage diseases by tagging apolipoproteins and/or the nanolipoproteins with appropriate targeting signals. Additional refinement is needed to extend the half-life of drug loaded lipoproteins by decreasing susceptibility to proteases and to control dynamic exchange with plasma or CNS lipoproteins perhaps by caging them to decrease the dissociation rate of the apolipoproteins and to control drug release. With the merits outweighing the challenges and shortcomings associated with using apolipoprotein-based nanostructures in nanomedicine and nanobiotechnology, their potential and scope appear very promising in the near future.

## Figures and Tables

**Table 1:** Apolipoproteins used in nanoparticles and their potential applications.

Apolipoprotein	Lipoprotein Association in Plasma or ^1^CNS	Physiological Function	Potential Applications
^2^ApoAI	^3^HDL, chylomicrons, ^3^VLDL	Structural protein in HDL; ^4^LCAT activator; promotes cholesterol efflux; binds ^4^SR-B1	Anti-atherogenic and drug delivery [39,40,41,42,43,44,45], siRNA delivery [46], imaging agent delivery [47,48]
ApoAII	HDL, chylomicrons, VLDL	Structural protein in HDL; activates hepatic lipase	Drug delivery [49]
ApoB-100	VLDL, ^3^IDL, ^3^LDL	Structural protein in VLDL, IDL, LDL; ligand for LDL receptor	Drug and imaging agent delivery [50,51,52,53,54,55,56,57,58,59,60,61,62]
ApoCII	Chylomicrons, VLDL, HDL	^4^Lipoprotein lipase activator	Drug delivery [49]
ApoE3	Chylomicron remnants, VLDL, IDL, HDL	Ligand for ^4^LDLr family of proteins; LCAT activator; antioxidant; promotes cholesterol efflux; binds SR-B1	Anti-atherogenic, drug, flavonoid and imaging agent delivery [18,39,63,64]
ApoJ (clusterin)	HDL	Anti-amyloidogenic; chaperone; antioxidant	Reduce aggregation of Aβ [65,66]

^1^ Central nervous system (CNS); ^2^ Apolipoprotein (apo); ^3^ High-density lipoproteins (HDL), intermediate density lipoprotein (IDL), low-density lipoprotein (LDL), very low-density lipoprotein (VLDL); ^4^ Lecithin cholesterol acyltransferase (LCAT); lipoprotein lipase (LPL); LDL receptor (LDLr); scavenger receptor class B type 1 (SR-B1).

**Table 2:** Summary and perspectives of synthesis of apolipoprotein-based nanoparticles.

Apolipoprotein	Nanoparticle Type	Nanoparticle Preparation Methodology	Comments and Perspectives
ApoAI	ApoAI–rHDL (method 1)	Thin film hydration of cargo–lipid complex followed by co-sonication with apoAI [169,170]	Conventional method of preparing rHDL
	ApoAI–rHDL (method 2)	Thin film hydration of cargo–lipid–protein complex followed by cholate dialysis [46,162,163]	Preparation of rHDL containing phospholipids with lower *T*_m_; presence of residual detergent a potential source of toxicity
	ApoAI–AuNP HDL (method 1)	Incubation of thiolated lipids and apoAI with AuNP [77,166]	Covalent conjugation of protein and lipids onto Au surface Circumvents apolipoprotein exchange
	ApoAI–AuNP HDL (method 2)	Nanoprecipitation to form Au–lipid NP, followed by co-sonication with apoAI [165]	Noncovalent interactions between protein and NP, allowing retention of native interactions
	ApoAI-coated HSA	Desolvation to form HSA NP, followed by conjugation to thiolated apoAI [133]	Non-lipidated formulation that allows trafficking of NP to the brain, possibly through SR-B1 on brain endothelial cells
	μHDL	Controlled mixing of lipids, payloads, and apoAI using a microfluidics device [164]	Suitable for scaled-up production of HDL as drug delivery vehicles
ApoAII	ApoAII–poly(butyl cyanoacrylate (PBCA)	Incubation of apoAII with PBCA NP, with or without polysorbate 80 [49]	Lipid-free polymeric formulation of apoAII
ApoB-100	ApoB-100-rLDL	Plasma-derived LDL used to reconstitute with therapeutic agents directly [50,51,52,53,54,55]	Templated approach that directly incorporates drug molecules with LDL
	ApoB-100 coated HSA	Desolvation to form HSA NP, followed by conjugation to thiolated apoB-100 [133]	Non-lipidated formulation that allows trafficking of NP to the brain, possibly through LDLr and LRP1 on brain endothelial cells
	ApoB–AuNP HDL	Nanoprecipitation to form Au–lipid NP, followed by co-sonication with apoB-100 [61]	Noncovalent interactions between protein and NP, allowing retention of native interactions
	ApoB–PBCA	Incubation of apoB with PBCA NP, with or without polysorbate 80 [49]	Lipid-free polymeric formulation of apoB; capable of crossing the BBB
	Dextran coated LDL	Incubation of dextran with LDL [73]	Polymeric formulation of drug/LDL complex; decreases exchange with other serum apolipoproteins
	Dendrimer coated LDL	Conjugation of G5.0 PPI dendrimers to LDL via –OH to –NH_2_ linkage [74]	Dendrimer grafting allows drug loading and controlled release on the surface of LDL
ApoCII	ApoCII–PBCA	Incubation of apoCII with PBCA NP, with or without polysorbate 80 [49]	Lipid free polymeric formulation of apoCII
ApoE3	ApoE3–rHDL	Thin film hydration of cargo–lipid complex followed by co-sonication with apoE3 [18,39,63,171]	Conventional method of preparing rHDL
	ApoE3–AuNP HDL	Thin film hydration of AuNP–lipid complex followed by co-sonication with apoE3 [64,172]	Noncovalent interactions between protein and NP, allowing retention of native interactions
	ApoE3 coated HSA	Desolvation to form HSA NP, followed by conjugation with apoE3 [130,131,132,134]	Non-lipidated formulation that allows trafficking of NP to the brain, possibly through LDLr and LRP1 on brain endothelial cells
	ApoE3–PBCA	Incubation of apoE3 with PBCA NP, with or without polysorbate 80 [49]	Lipid-free polymeric formulation of apoE3; capable of crossing the BBB
ApoJ	ApoJ–PBCA	Incubation of apoJ with PBCA NP, with or without polysorbate 80 [49]	Lipid-free polymeric formulation of apoJ
